# hARACNe: improving the accuracy of regulatory model reverse engineering via higher-order data processing inequality tests

**DOI:** 10.1098/rsfs.2013.0011

**Published:** 2013-08-06

**Authors:** In Sock Jang, Adam Margolin, Andrea Califano

**Affiliations:** 1 Sage Bionetworks, 1100 Fairview Avenue North, Seattle, WA 98109, USA; 2 Department of Systems Biology, Biochemistry and Molecular Biophysics, Biomedical Informatics, and Herbert Irving Comprehensive Cancer Center, Columbia University Medical Center, New York, NY 10032, USA

**Keywords:** ARACNe, higher-order data processing inequality, information theory, transcriptional regulatory network, reverse engineering

## Abstract

A key goal of systems biology is to elucidate molecular mechanisms associated with physiologic and pathologic phenotypes based on the systematic and genome-wide understanding of cell context-specific molecular interaction models. To this end, reverse engineering approaches have been used to systematically dissect regulatory interactions in a specific tissue, based on the availability of large molecular profile datasets, thus improving our mechanistic understanding of complex diseases, such as cancer. In this paper, we introduce high-order Algorithm for the Reconstruction of Accurate Cellular Network (hARACNe), an extension of the ARACNe algorithm for the dissection of transcriptional regulatory networks. ARACNe uses the data processing inequality (DPI), from information theory, to detect and prune indirect interactions that are unlikely to be mediated by an actual physical interaction. Whereas ARACNe considers only first-order indirect interactions, i.e. those mediated by only one extra regulator, hARACNe considers a generalized form of indirect interactions via two, three or more other regulators. We show that use of higher-order DPI resulted in significantly improved performance, based on transcription factor (TF)-specific ChIP-chip data, as well as on gene expression profile following RNAi-mediated TF silencing.

## 1. Introduction

Cellular phenotypes are determined by a complex web of physical interactions between gene products [1]. Modelling these relationships helps to organize the list of parts encoded in the genome into functional genetic networks, a crucial step towards the understanding of mechanisms contributing to normal cell physiology as well as of their dysregulation in disease.

With the advent of high-throughput technologies, a large amount of molecular profile data have been generated from large numbers of samples associated with a variety of diseases. Through use of reverse engineering algorithms, these data have shown great promise in the dissection of transcriptional regulatory networks on a genomic scale [2–10]. While the caveats associated with the use of gene expression data for transcriptional network inference have been well documented and studied throughout the past decade [11], a preponderance of studies and high-impact discoveries in systems biology have established such approaches as widely accepted tools in a systems biologist's arsenal [6,12–18]. Reverse engineering approaches have been developed using the mathematical frameworks established in disciplines such as Bayesian networks [4,5,19], dynamical systems [8] and information theory [2,6,7,11–13,16,17,20]. For instance, the relevance network approach [2,21], one of the earliest proposed, assumes that genes co-expressed above statistical significance are more likely to represent regulatory interactions. Unfortunately, owing to long chains of regulatory events, a large number of gene pairs may be co-expressed without necessarily implicating a physical interaction. Thus, relevance networks, whether based on mutual information or Spearman correlation, typically generate a large number of false positives.

A survey of such approaches and their relative merits is reviewed in [11] and are outside the scope of this article. Rather, the current study is intended to build on and improve the body of research associated with the information theoretic algorithm ARACNe (Algorithm for the Reconstruction of Accurate Cellular Network), which has emerged as a widely referenced approach that has been experimentally validated in numerous applications, leading to key biological discoveries [6,12–14].

ARACNe was developed to maintain the simplicity of relevance networks, while using rigorous information theoretic principles to eliminate the vast majority of indirect interactions (i.e. false positives). By limiting interactions only to pairs where at least one of the genes is a transcription factor (TF), ARACNe also addressed the issue of interaction directionality, since a TF can regulate a non-TF but the opposite is not true. Thus, the only undirected interactions in ARACNe networks are those between two TFs, where directionality cannot be disambiguated.

To eliminate indirect interactions, ARACNe uses the data processing inequality (DPI) theorem, from information theory, stating that information transferred directly (i.e. through a physical interaction) is always larger than information transferred indirectly, i.e. via an intermediary. Here, information is formally computed by the mutual information, *I*(*x*;*y*) = *S*(*x*) + *S*(*y*) − *S*(*x*, *y*), where *S*(*u*) represents the entropy of the variable *u*. We note that the DPI does not apply for other measures, such as Spearman correlation, unless interactions are linear, which is clearly not the case in biological systems. Experimental validation shows that the DPI removes a vast majority of false positive interactions, leading to highly accurate regulatory models [6,7,20,22].

Given its simplicity and the fact that it could consider each interaction independently, ARACNe was the first reverse engineering algorithm to successfully scale up to the complex regulatory networks of mammalian cells. For instance, 90 per cent of ARACNe-inferred and experimentally tested MYC targets in human B cells were validated as directly regulated by MYC [6], validation of targets in high-grade human glioma confirmed 40 out of 50 tested interactions for the transcription factors C/EBPβ, Stat3, RUNX1 and BHLHB2 [12], and the algorithm could correctly dissect a large number of transcriptional interactions involved in the synergistic control of germinal centre B cell proliferative programmes by FOXM1 and MYB [14,23,24]. More importantly, interrogation of ARACNe-inferred regulatory networks has allowed elucidation of key drivers of normal and disease-related phenotypes [6,12,13,15].

In this study, we explore improvements based on the nested iterative application of higher-order DPI tests [25,26]. The resulting algorithm, hARACNe (higher-order ARACNe), is thus designed to identify and remove a significant number of false positive interactions that could not be identified by first-order DPI (DPI^1^) analysis. Use of higher-order DPI analysis does not affect the generality of the method and is applicable to any network analysis, including those using static, time-course and even post-translationally predicted data [7,27].

Our analysis shows that hARACNe can systematically eliminate false positive interactions that were missed by DPI^1^ logic of ARACNe, thus significantly improving inferred TF–target interaction accuracy, based on MYC-binding data from ChIP-chip data [6,28] as well as gene expression profile (GEP) analysis following RNAi-mediated silencing of BCL6 [16]. Thus, our data show that hARACNe constitutes an advance in the identification of bona fide TF–target interactions of biological relevance.

## 2. Material and methods

### 2.1. Data sources

For this study, 254 previously published [6,17,20] GEPs were used, representing 17 distinct normal and tumour-related B-cell phenotypes from primary patient biopsies and tumour-derived cell lines. In addition, we used a set of 226 previously published [16] GEPs from human B-cell lymphoma, including normal samples from naive, memory and germinal centre B cells isolated from human tonsils and patient derived tumour samples, including diffused large B-cell lymphoma (DLBCL), follicular lymphoma and chronic lymphocytic leukaemia (CLL). The first dataset was profiled using the HG-U95Av2 GeneChip platform, whereas the second set was profiled using the HG-U133 Plus2.0 GeneChip (Affymetrix) [6,7,14,16,17,20]. All profiles discussed in this research are accessible from the Gene Expression Omnibus (GEO; National Center for Biotechnology Information), through GEO series accession nos. GSE2350 and GSE12195. The list of phenotypes is found in table 1.

**Table 1.** Human B-cell phenotypes in the two GEPs.

HG-U95 Av2		HG-U133 Plus2.0	
cell type	no.	cell type	no.
diffuse large B-cell lymphoma	68	naive B cell	5
Burkitt's lymphoma	33	memory B cell	5
follicular lymphoma	14	germinal centre B cell	11
mantle cell lymphoma	8	B-cell chronic lymphocytic leukaemia	16
B-cell chronic lymphocytic leukaemia	34	follicular lymphoma	38
hairy cell leukaemia	16	diffuse large B-cell lymphoma	128
multiple myeloma	4	undefined	23
Hodgkin's lymphoma	4		
primary effusion lymphoma	9		
splenic lymphoma with villous lymphocytes	12		
large cell lymphomas	5		
Burkitt's lymphoma type III	3		
undefined	2		
germinal centre B cells	17		
naive B cell	5		
memory B cells	5		
cord blood	5		
total	254	total	226

Ramos and Mutu (human Burkitt's lymphoma) cell lines were analysed in ChIP-chip assays to identify the genes whose proximal promoter is bound the MYC protein. ChIP-chip significance analysis (CSA) was applied, where *p*-values were first derived for each probe from three replicate experiments, as described in [29]. Values of *p* were then integrated across a 500-base region surrounding the transcription start site of the gene, using a gamma cumulative distribution function. Each promoter was associated with the highest 500-base MYC-localization segment, and the false discovery rate (FDR) was computed using the Benjamini Hochberg procedure, as a function of gene rank. More precise procedures were described in [28].

In addition, DLBCL cell lines, including LY7, Pfeiffer and VAL, were profiled following lentivirus-mediated shRNA silencing of the BCL6 TF. The experimental procedures and conditions used to perform these experiments are described in [16]. Following BCL6 silencing, differentially expressed genes were identified (FDR < 0.05). ARACNE- and hARACNe-inferred BCL6 targets (BCL6 regulons) were then compared with differentially expressed genes.

For both the ChIP-chip and shRNA experiments, the procedures described earlier were used to infer a list of ‘positive’ TF–target interactions, which were compared with the predictions made by hARACNe and ARACNe to compute the number of true positives versus false positives removed by the additional pruning steps:
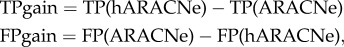


For comparison with ChIP-chip experiments, TP and FP are the number of ARACNe- or hARACNe-inferred MYC targets that were identified as bound and not bound by MYC, respectively. For comparison with shRNA experiments, TP and FP are the number of ARACNe- or hARACNe-inferred BCL6 targets that were identified as differentially regulated or not differentially regulated, respectively, following BCL6 inhibition. We note that the decrease in true positives due to removing interactions is equivalent to the increase in false negatives.

As with most ‘gold standards’ used in assessment of systems biology applications, we note that both the ChIP-chip and shRNA experiments are imperfect tests to identify true TF–target regulatory interactions. In particular, each assay only measures one (necessary but not sufficient) criterion of a regulatory interaction, and therefore may over-estimate the number of interactions. Moreover, each assay is performed in only a subset of tumour sub-types that were profiled in the microarray dataset, and therefore may underestimate the number of interactions, as only those active in the tested sub-types will be detected. Thus, although the exact number of true and false positives reported for each method are inexact, the TFgain and FPgain statistics should represent fair tests of the relative performance of each method. Moreover, the ChIP-chip and shRNA experiments measure different aspects of TF–target interactions and were performed on different TFs; therefore, consistent performance increases in comparison with these two distinct experiments lend strong support to the benefit of a method.

### 2.2. hARACNe algorithm design

**Figure 1.**
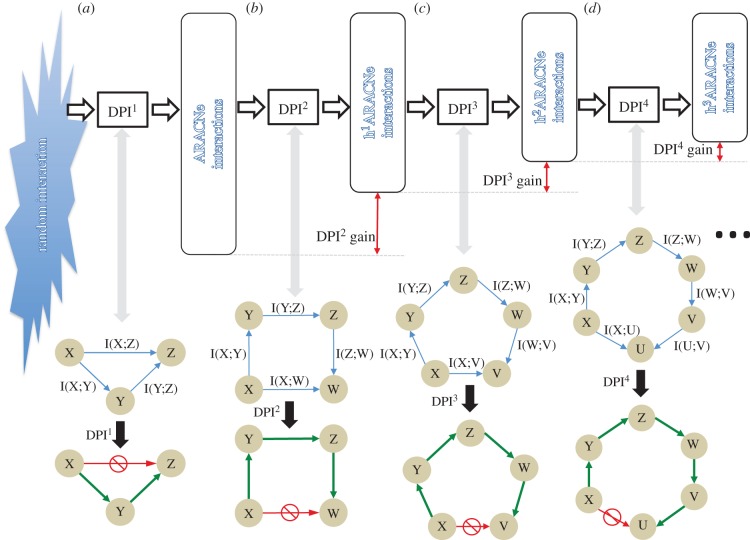
(*a*) Pruning process by DPI in triplet, (*b*) DPI^2^ nested iterative procedure to detect indirect interaction in quadruplet, (*c*) DPI^3^ sequential procedure to detect indirect interaction in quintuplet, and (*d*) DPI^4^ sequential procedure to detect indirect interaction in sextuplet.

The DPI is a simple but powerful theorem that starts from the simple axiom that as information is transferred through a lossy network, it can only be reduced and never increased. A Markov chain is at the heart of the DPI concept. A Markov chain is a linear sequence of states such that knowledge of the state at position *i*_0_ makes all states at position *i* > *i*_0_ independent of states at position *i* < *i*_0_. In other words, any state provides all the necessary information to infer downstream states in the chain [26,30]. In a gene regulatory network, a Markov chain may represent a sequence of regulatory interactions *R*_1_ → *R*_2_ → ⋯ → *R_N_*. In this case, DPI^1^ analysis can remove indirect interactions of the type *R_i_* → *R_i_*_+2_, where a distinct Markov state *R*_*i*+1_ exists that makes *R_i_*_+2_ independent of *R_i_* (figure 1*a*). In this case, the DPI states that, if any information is lost through interactions (an obvious true statement for any biological regulatory cascade), then *I*(*R_i_*; *R_i_*_+2_) is strictly smaller than both *I*(*R_i_*; *R_i_*_+1_) and *I*(*R_i_*_+1_; *R_i_*_+2_), i.e.2.1
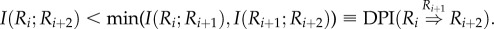
This means that the *R_i_* → *R_i_*_+2_ interaction is removed because it is indirect through *R_i_*_+1_.

To extend DPI^1^ to higher-order DPIs, consider a Markovian quadruplet *R_i_* → *R*_*i*+1_ → *R*_*i*+2_ → *R*_*i*+3_. Then, the second-order DPI (DPI^2^) could be expressed as follows:2.2
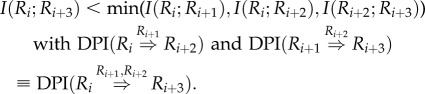
Or, in other words, the *R_i_* → *R*_*i*+3_ interaction is removed as indirect through *R*_*i*+1_ and *R*_*i*+2_.

*Proof*. It follows from the Markov property satisfying conditional independency among a Markov triplet as *I*(*R_i_*;*R_i_*_+3_*|R_i_*_+1_) = *I*(*R_i_*_+1_;*R_i_*_+3_*|R_i_*_+2_) = 0. Therefore, by repeatedly using the chain rule, *I*(*R_i_*;*R_i_*_+1_, *R_i_*_+2_) = *I*(*R_i_*;*R_i_*_+1_) + *I*(*R_i_*;*R_i_*_+2_*|R_i_*_+1_), we obtain this inequality as follows:
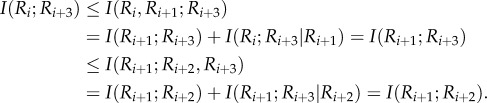


Figure 1*b* shows graphically how the DPI^2^ can be nested iteratively applied to identify additional false positive interactions within a Markov quadruplet.

Next, consider a Markovian chain of any length *n* + 2, *R_i_* → *R*_*i*+1_ → ⋯ → *R_i+n_* → *R*_*i*+*n*+1_. We can apply the *n*th-order DPI (DPI*^n^*) with conditional independence among any sub-chain as follows:2.3
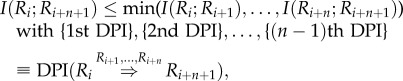
where {*k*th DPI}means the super set of all possible combinations of *k*th-order DPI. Figure 1*c*,*d*, for instance, show examples for DPI^3^ and DPI^4^.

Higher-order DPI pruning process is always sequentially conducted after lower-order DPI is processed in advance, and pairwise mutual information between TFs and TGs are once computed in original ARACNe (DPI^1^), and we iteratively prune indirect interactions from lower DPI to higher DPI. Thus, *k*th-order DPI does not need to revisit lower-order DPI application. This sequential pruning with higher-order DPIs may be applicable to any modified versions of ARACNe, which uses DPI^1^ to prune indirect interactions.

## 3. Results

### 3.1. Reverse engineered transcriptional networks comparing ARACNe and hARACNe

The number of transcriptional interactions in the reconstructed networks was compared, and the distribution of TF targets is shown in table 2. In the human B-cell data profiled with the Affymetrix U95 platform, there were 12 600 probe IDs, of which 1225 corresponded to TFs, representing 848 unique genes. ARACNe identified 155 526 transcriptional interactions at the probe ID level. After applying DPI^2^, 134 452 interactions were left and 21 074 were removed as indirect interactions. When DPI^3^ was applied, 132 018 interactions were left and an additional 2434 were removed as indirect. Analysis with fourth- and fifth-order DPI removed only 280 and no interaction, respectively. Thus, it appears that high-order DPI reaches saturation rapidly and that only DPI^2^ and DPI^3^ provide substantial false positive filtering power. Yet, high-order DPI, DPI^4^ and above, have relatively high computational cost compared with efficiency of filtering power. With the second B-cell dataset, profiled using the Affymetrix U133 platform, there were 14 090 probe IDs, of which 1290 were TFs representing 1209 unique genes. ARACNe identified 198 766 transcriptional interactions at the probe ID level. DPI^2^ produced 142 037 interactions, with 56 729 removed as indirect interactions. DPI^3^ produced 138 697 interactions, with 3340 additional ones removed as indirect interactions. Finally, DPI^4^ removed an additional 1533 interactions as indirect. We did not apply DPI of order greater than four.

**Table 2.** The number of interactions: comparisons with the adaptive partitioning method with nested iterative application of higher-order DPIs. The number of interactions inferred by ARACNe and hARACNe. Mutual information values were computed using the adaptive partitioning method [31]. See http://wiki.c2b2.columbia.edu/califanolab/index.php/Software/ARACNE.

adaptive partitioning	HG-U95 Av2	HG-U133 Plus2.0
ARACNe	155 526	198 766
h^1^ARACNe	134 452	142 037
h^2^ARACNe	132 018	138 697
h^3^ARACNe	131 738	137 164

**Figure 2.**
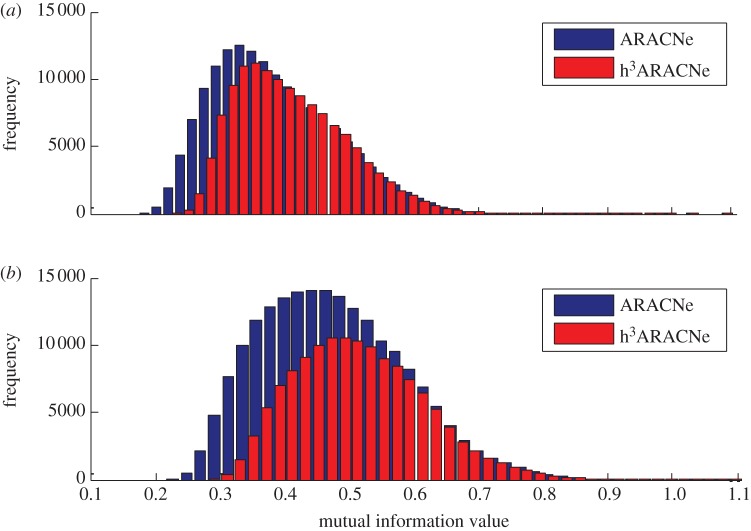
Overall mutual information comparison between original ARACNe and h^3^ARACNe networks. (*a*) U95 Av2 distribution change and (*b*) U133 Plus2 distribution change.

**Figure 3.**
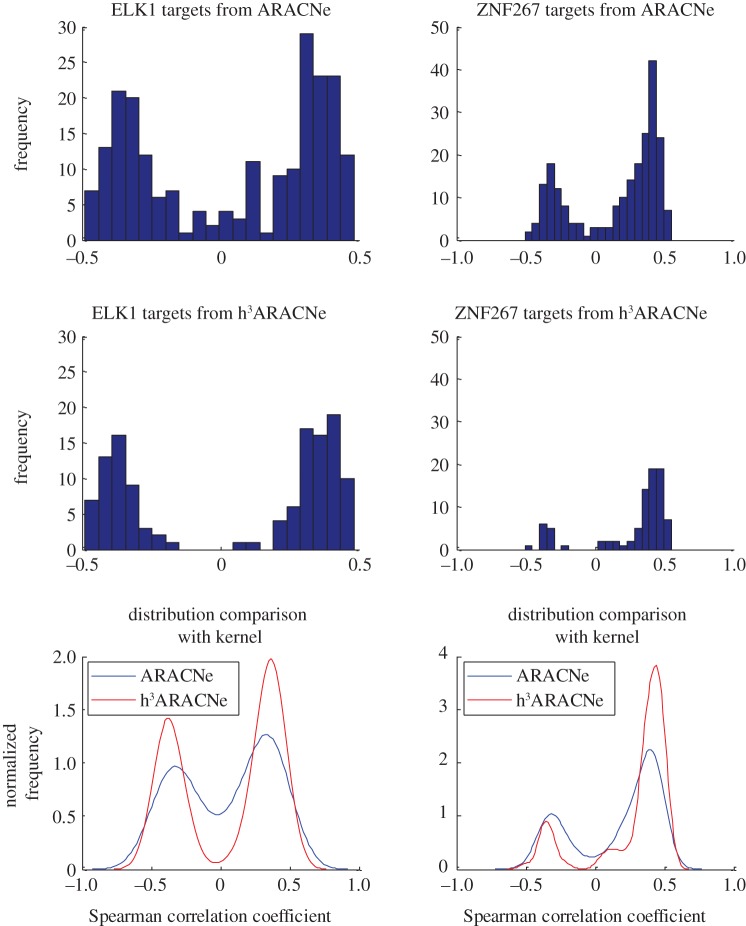
Correlation distribution of the most differential TF targets between original ARACNe and h^3^ARACNe networks.

In our comparative analyses, every TF had a different number of ARACNe- and hARACNe-inferred transcriptional targets. The distributions of transcriptional targets for ARACNe and hARACNe in the human B-cell analysis are shown in figure 2. For instance, from the U95 dataset, the ELK1 TF has 233 ARACNe-inferred targets, and we randomly selected ELK1 among the TFs which have more than 200 targets in ARACNe. Of these, 97 were removed by DPI^2^, leaving only 136 targets (figure 3). However, DPI^3^ removed only seven additional targets and no additional targets were removed by DPI^4^. An identical pattern was observed in the U133 human B-cell dataset analysis. Among all TFs, ZNF267 had the largest number of targets removed by higher-order DPI analysis. Of 223 ARACNe-inferred targets, 121 were removed by DPI^2^, and 30 and 5 additional targets were removed by DPI^3^ and DPI^4^, respectively, leaving only 86 targets.

As described, hARACNe removed a significant percentage of interactions inferred by ARACNe. This may improve the accuracy of inferred networks if the filtered interactions were enriched in false positives (i.e. indirect interactions). To test this hypothesis, we compared the ARACNe and hARACNe predictions against two datasets providing orthogonal evidence of TF–target interactions (see figure 2 for more details).

The first dataset was generated through ChIP-chip experiments designed to detect direct biochemical interactions between the MYC proto-oncogene and genome-wide promoter regions. The second dataset used RNAi experiments to detect genes differentially regulated upon inhibition of BCL6. We used data related to MYC and BCL6 based on availability of datasets and based on their central role as proto-oncogene B-cell leukaemias and lymphomas.

### 3.2. ChIP-chip functional validation of MYC targets

We first compared ARACNe- and hARACNe-inferred targets of the MYC TF against genes with experimentally assessed interactions between the MYC protein and their proximal promoter regions. The assessment was based on existing ChIP-chip assays in Ramos and Mutu (human Burkitt's lymphoma) cell lines [29]. Based on the CSA algorithm [28], 5307 and 3310 putative MYC-binding targets could be predicted in Ramos and Mutu cell lines, respectively. Comparing ARACNe- and hARACNe-inferred MYC regulons, from the U95Av2 data, 18 interactions were removed by hARACNe. Based on Ramos cell assays, 13 of these 18 interactions (hypergeometric test: *p*-value = 0.0178) were identified as false positives (i.e. no detected interaction between MYC and the gene's promoter region) and only five were identified as true positives (table 3). Based on Mutu cell assays, 14 of the 18 interactions were identified as false positives, and only four of 18 were identified as true positives (hypergeometric test: *p*-value = 2.8713×10^−7^).

**Table 3.** (*a*) ChIP-chip experimental validation with Ramos and Mutu cell lines for MYC targets and (*b*) hypergeometric test in order to check if the gain is significantly derived from FPs.

(*a*)								
MYC: ChIP-chip		Ramos			MYC: ChIP-chip	Mutu		
		TP	FP	Fisher's exact test *p*-value of inferred interactions		TP	FP	Fisher's exact test *p*-value of inferred interactions
HG-U95Av2								
DPI^1^		214	258	0.1428	DPI^1^	170	302	3.09×10^−6^
DPI^2^		210	249	0.1077	DPI^2^	167	292	1.85×10^−6^
DPI^3^		209	246	0.09444	DPI^3^	166	289	1.63×10^−6^
DPI^4^		209	245	0.08747	DPI^4^	166	288	1.40×10^−6^
gain		5	13	n.a.	gain	4	14	n.a.
(*b*)								
HG-U95Av2	total gain between DPI^1^ and DPI^4^(nested)	Ramos			total gain between DPI^1^ and DPI^4^(nested)	Mutu		
		TP	FP	hypergeometric test *p*-value		TP	FP	hypergeometric test *p*-value
	18	5	13	0.01779333	18	4	14	2.87×10^−4^

As a result, it appears that hARACNe was three times more likely to remove false interactions not supported by a corresponding MYC-binding site in the proximal promoter region than to remove interactions with target whose promoter was bound by MYC.

### 3.3. Differentially expressed genes following shRNA-mediated BCL6 silencing

To further check whether the additional pruning produced by higher-order DPI improved the accuracy of TF–target prdiction, we performed lentivirus-mediated shRNA silencing of the BCL6 gene and tested the enrichment of differentially expressed genes in ARACNe- versus hARACNe-inferred BCL6 targets. GEPs were measured using the Affymetrix HG-U95A GeneChip platform with DLBCL and CLL cell lines (LY7, Pfeiffer and VAL cell lines), where cells were infected with control non-target shRNA or validated shRNA targeting BCL6. Differentially expressed genes were identified using fold-change criteria and a 0.05 FDR threshold using a non-parametric *U*-test. In LY7, Pfeiffer and VAL cell lines, 1507, 3706 and 3199 differentially expressed genes were identified, respectively. From LY7 data, out of 334 ARACNe-inferred BCL6 targets, 318 were identified by sequential application of DPI^2^, DPI^3^ and DPI^4^. Twenty gains were achieved by hARACNe. Eighteen out of 20 were not differentially expressed following BCL6 silencing and may thus be considered false positives. Conversely, two BCL6 targets were newly identified due to consensus scoring analysis followed by 100 bootstrappings (hypergeometric test: *p*-value = 5.5955×10^−14^), which were differentially expressed following BCL6 knockdown and may have been considered true positives (table 4). In Pfeiffer cells, all 16 interactions removed by hARACNe(nested iterative procedures of DPI^2^, DPI^3^ and DPI^4^) were identified as false positives and none were identified as true positives (hypergeometric test: *p*-value = 8.6441×10^−7^). Finally, in VAL cells, all 16 interactions removed by hARACNe(nested iterative procedures of DPI^2^, DPI^3^ and DPI^4^) were also identified as false positives and none were identified as true positives (hypergeometric test: *p*-value = 2.5294×10^−8^). Tables 3 and 4 also include the statistical analysis (Fisher's exact test) with TPs and FPs from experimentally validated targets with each step of hARACNe-inferred targets in order to show how significantly targets were identified by either ARACNe or each cumulative order of hARACNe.

**Table 4.** (*a*) Gain and loss BCL6 targets comparison between ARACNe and hARACNe by shRNA library and (*b*) hypergeometric test in order to check if the gain is significantly derived from FPs.

(*a*)												
BCL6: shRNA library		LY7			BCL6: shRNA library	Pfeiffer			BCL6: shRNA library	VAL		
		TP	FP	Fisher's exact test *p*-value of inferred interactions		TP	FP	Fisher's exact test *p*-value of inferred interactions		TP	FP	Fisher's exact test *p*-value of inferred interactions
HG-U95Av2												
DPI^1^		54	280	0.0136	DPI^1^	144	190	1.06×10^−7^	DPI^1^	117	217	6.97×10^−5^
DPI^2^		57	265	0.001614	DPI^2^	146	176	1.60×10^−9^	DPI^2^	118	204	6.32×10^−6^
DPI^3^		56	262	0.001988	DPI^3^	144	174	2.28×10^−9^	DPI^3^	117	201	5.54×10^−6^
DPI^4^		56	262	0.001988	DPI^4^	144	174	2.28×10^−9^	DPI^4^	117	201	5.54×10^−6^
gain		2	18	n.a.	gain	0	16	n.a.	gain	0	16	n.a.
(*b*)												
HG-U95Av2	total gain between DPI^1^ and DPI^4^(nested)	LY7			total gain between DPI^1^ and DPI^4^(nested)	Pfeiffer			total gain between DPI^1^ and DPI^4^(nested)	VAL		
		TP	FP	hypergeometric test p-value		TP	FP	hypergeometric test *p*-value		TP	FP	hypergeometric test *p*-value
	20	2	18	5.60×10^−14^	16	0	16	8.64×10^−7^	16	0	16	2.53×10^−8^

## 4. Discussion

The goal of this work was to improve the widely used ARACNe algorithm by further reducing false positive interactions, thus leading to more accurate inference of interaction networks. We note that our work is not intended to address other foundational issues of reverse engineering approaches or of the ARACNe algorithm, such as the assumptions inherent to the use of mRNA data. Rather, we use the widely accepted ARACNe assumptions as a starting point and assess the ability of higher-order DPI analysis to improve prediction accuracy based on theoretical arguments and comparison with independent ‘gold standard’ datasets. Within this limited context, we believe that this work represents a useful contribution to the field, based on the widespread use of ARACNe and the benefit of an extension improving its accuracy.

From a theoretical standpoint, we note that higher-order, indirect Markov chain interactions detected by hARACNe would also be eliminated by repeated application of the DPI^1^, under certain assumptions. Specifically, these would require that (i) the network have a tree (or locally tree-like) structure, (ii) the network contain only pairwise interactions, and (iii) mutual information be measured without errors. Given that each of these assumptions may be individually violated and given the large number of potential pairwise interactions, higher-order DPIs provide additional filters to increase the global Markov chain stringency in ARACNe-inferred networks, thus eliminating indirect interactions that may have been missed by DPI^1^. In this context, the experimental data we have provided, demonstrating a higher accuracy of hARACNe networks, represents the most meaningful test of the method.

Yet, there are a few computational limitations in hARACNe. First, since hARACNe starts from an ARACNe-inferred network, on which it nested iteratively applies higher-order DPI analyses, it must consider a large number of candidate Markov chain paths, traversing up to four interactions, and is thus computationally intensive. Our results, however, show that DPI^2^ provides the greatest pruning effect while higher-order DPIs have a significantly lower detection rate. Thus, one may want to consider whether to apply only DPI^2^ or up to DPI^3^ to obtain the highest increase in accuracy at the lowest computational cost.

Our experimental design for evaluating hARACNe relied on two datasets providing orthogonal evidence of direct TF–target interactions. Specifically, we used ChIP-chip assays and shRNA-mediated silencing to show that hARACNe preferentially removes false positive interactions. In BCL6 silencing experiments, interactions removed by hARACNe had significantly more false positives than true positives. Similarly, when considering ChIP-chip experimental data on MYC-binding sites, a similar improvement in the false positive predictions was introduced by hARACNe. Overall, the higher-order DPI analysis inferred a more reliable network, compared with the DPI^1^ procedure of the original ARACNe algorithm.
